# Plaque distribution of low-grade basilar artery atherosclerosis and its clinical relevance

**DOI:** 10.1186/s12883-016-0785-y

**Published:** 2017-01-10

**Authors:** Jin Yu, Ming-Li Li, Yu-Yuan Xu, Shi-Wen Wu, Min Lou, Xue-Tao Mu, Feng Feng, Shan Gao, Wei-Hai Xu

**Affiliations:** 1 Department of Neurology and Radiology, Peking Union Medical College Hospital, Chinese Academy of Medical Sciences, Shuaifuyuan1, Dongcheng District, Beijing, 100730 China; 2 Department of Neurology and Radiology, General Hospital of Chinese People’s Armed Police Force, Beijing, China; 3 Department of Neurology, Zhejiang University 2nd affiliate hospital, Hangzhou Shi, Zhejiang Province China

**Keywords:** Intracranial atherosclerosis, Clinical research, Plaque, Magnetic resonance imaging

## Abstract

**Background:**

The underlying pathophysiology of BA distribution is unclear and intriguing. Using high-resolution magnetic resonance imaging (HR-MRI), we sought to explore the plaque distribution of low-grade basilar artery (BA) atherosclerosis and its clinical relevance.

**Methods:**

We retrospectively analyzed the imaging and clinical data of 61 patients with low-grade atherosclerotic BA stenosis (<50%). On HR-MRI, the plaques were categorized based on the involvement of the ventral, dorsal, or lateral sides of BA wall. A culprit plaque was defined if it was on the same slice or neighboring slices of symptomatic pontine infarcts and played a probable causal role (dorsal plaques with median pontine infarcts or lateral plaques with ipsilateral pontine infarcts). The relationships between plaque distribution and clinical presentations were analyzed.

**Results:**

Twenty-five symptomatic and thirty-six asymptomatic BAs with 752 HR-MRI image slices were studied. The average length of BA atherosclerosis plaques was 12.16 ± 5.61mm (10.30 ± 6.44mm in symptomatic and 13.46 ± 7.03mm in asymptomatic patients, *p* = 0.079). The plaque distribution was similar at ventral (29.0%), dorsal (37.6%) and lateral walls (33.1%). The BA plaques in symptomatic patients were more frequently located at the dorsal (42.5%) and lateral (41.2%) walls than at the ventral walls (16.1%; *P* < 0.05). Compared with symptomatic patients, asymptomatic patients more likely had their plaques distributed at the ventral walls (*P* = 0.022). Culprit plaques were observed in 85.0% (17/20) pontine infarcts in symptomatic patients and only 14.3% (2/14) silent pontine infarcts in asymptomatic patients (*p* < 0.001).

**Conclusions:**

Low-grade BA atherosclerosis has a long distribution and evenly involves ventral, dorsal and lateral walls. The plaques at dorsal and lateral walls are associated with symptomatic pontine infarcts but not with silent infarcts.

**Electronic supplementary material:**

The online version of this article (doi:10.1186/s12883-016-0785-y) contains supplementary material, which is available to authorized users.

## Background

Emerging evidence has suggested the distribution of atherosclerotic plaque plays an important role in the occurrence of ischemic events [1]. In coronary artery and middle cerebral artery atherosclerosis, plaques naturally tend to form at the positions opposite to the orifices of branch or penetrating arteries [2, 3]. Once the plaques locate near the perforating orifices, they are more likely symptomatic and related to “snow plowing” effect during stenting process [3].

Basilar artery (BA) is the largest artery in the posterior circulation and forms the central core of this vascular territory. It gives rise to many side branches which can be divided into three groups: the cerebellar arteries, cerebral hemisphere branches and the perforating arteries [4]. The dorsal and lateral surfaces of the BA are a rich source of perforating arteries, while nearly no perforating arteries arise from the ventral surfaces of the BA [5]. To provide deep insight of the pathophysiology of BA atherosclerosis, in this study, we systematically described and compared the plaque distributions of symptomatic and asymptomatic low-grade BA atherosclerosis and clarify their clinical relevance.

## Methods

### Patients

This observational study was approved by the ethics committee at Peking Union Medical College Hospital, General Hospital of Chinese People’s Armed Police Forces, and Zhejiang University 2^nd^ affiliate hospital. We retrospectively reviewed the high-resolution magnetic resonance imaging (HR-MRI) databases (2006 to 2013) from three medical centers in China. All patients with BA atherosclerotic plaque on HR-MRI were enrolled if they fulfilled the following criteria: (1) low-grade BA atherosclerotic stenosis (<50%) were detected by magnetic resonance angiography; (2) one or more atherosclerotic risk factors including hypertension, hypercholesterolemia, diabetes mellitus, and cigarette smoking; (3) image quality good enough for analysis. Patients with the following conditions were excluded by clinical presentations, lab work and imaging:(1) arteritis; (2) dissection; (3) evidence of cardioembolism; and (4) co-existent vertebral artery atherosclerotic stenosis (≥50%).

Symptomatic patients were defined if there was an ischemic stroke in the distribution of BA within the proceeding four weeks, and new ischemic lesions were identified on diffusion-weighted and T2 weighted images. Asymptomatic patients were defined if there was no history of cerebrovascular events in the distribution of BA. Silent infarcts were defined if brain stem lesions were incidentally identified by MRI in clinical stroke-free subjects.

### Imaging protocol

A 3.0 T GE scanner (Twinspeed; GE Medical Systems) with an 8-channel head coil was used. Conventional 3 dimensional time-of-flight (3D TOF) magnetic resonance angiography was obtained in an axial plane with the following parameters: repetition time/echo time (TR/TE) = 25.0/3.1ms; flip angle = 20°; field of view(FOV) = 240mm × 240mm; matrix size = 384 × 224; slice thickness = 1.4 mm, without slice gap; 1 signal averages. To examine the BA wall, HR-MRI sequences including T2 weighted imaging (T2WI), T1 weighted imaging (T1WI) and proton density weighted imaging (PDWI) were performed. Eight to fourteen slices, depending on the length of BA, were acquired along the short axis of the BA using the double-oblique plane with the following parameters: T2WI (TR/TE = 3360/45.3ms; FOV = 130mm × 130mm; matrix size = 384 × 384; slice thickness = 2mm; slice gap = 0.5mm; scan times = 2min10s), T1WI (TR/TE = 840/11.4ms; FOV = 130mm × 130mm; matrix size = 384 × 384; slice thickness = 2mm; slice gap = 0.5mm; scan times = 1min38s), PDWI (TR/TE = 2440/19.7ms; FOV = 130mm × 130mm; matrix size = 384 × 384; slice thickness = 2mm; slice gap = 0.5mm; scan times = 2min53s); 4 signal averages. Voxelsize was 0.33 × 0.33 × 2 mm. No smoothing filter was applied.

### Image analysis

**Fig. 1:**
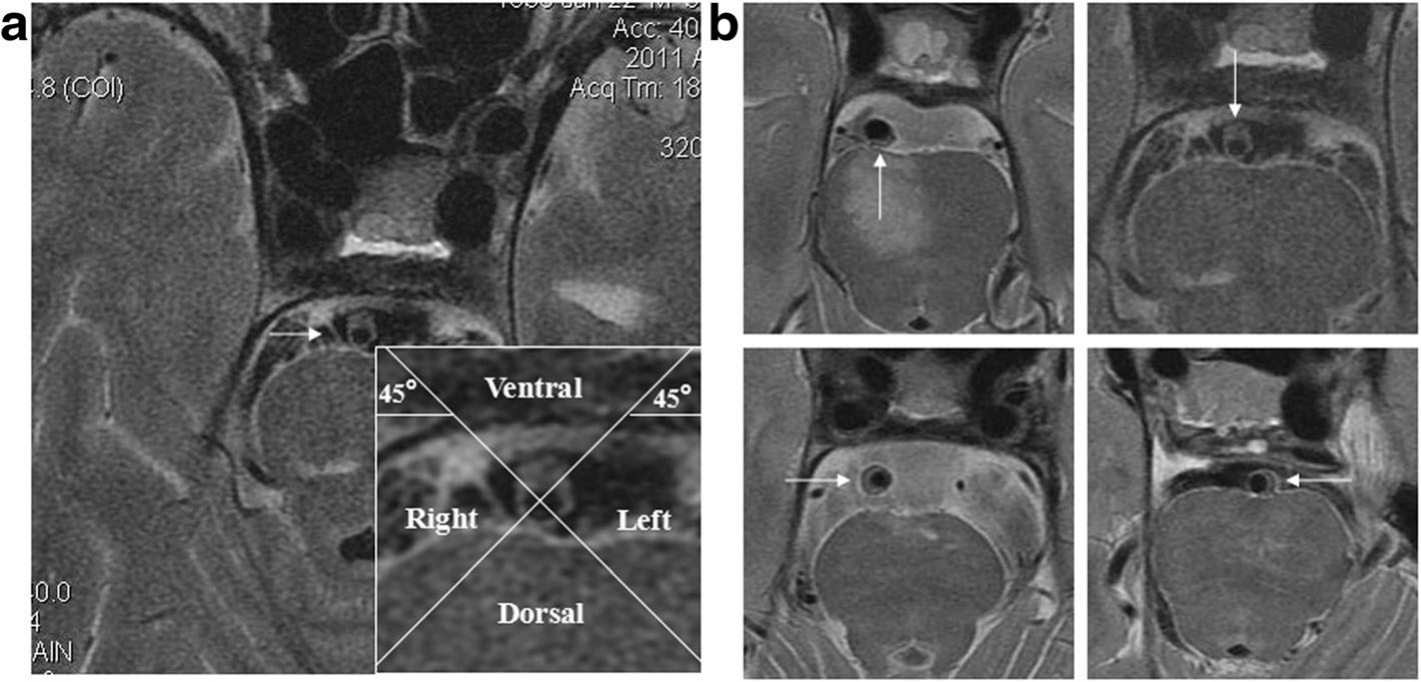
**a** An alignment grid to demonstrate how each cross-section is divided into 4 quadrants. **b** Examples of plaques involving the dorsal, ventral, and lateral (*right or left* ) walls, respectively. (T2WI)

**Fig. 2:**
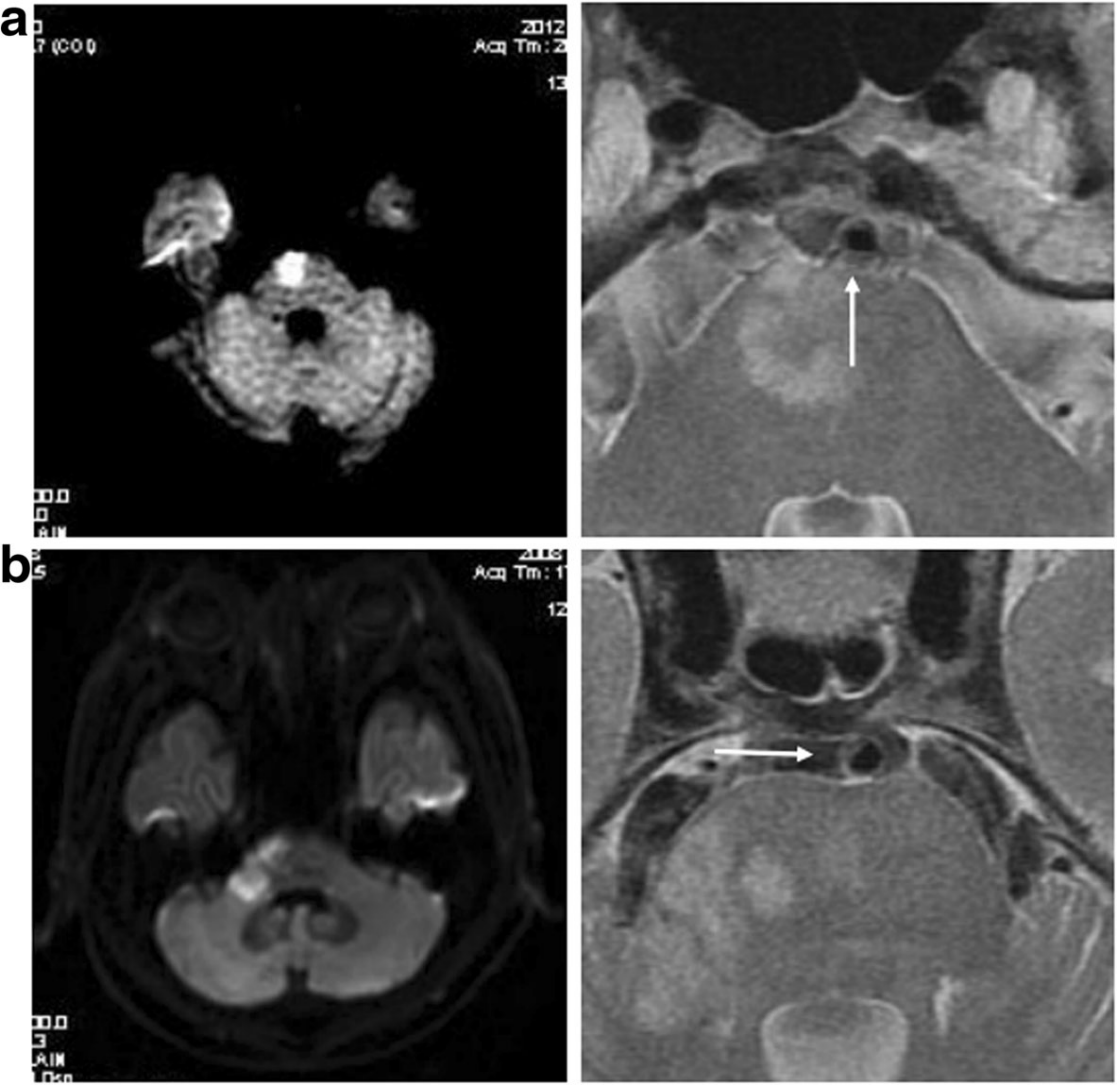
**a** a dorsal wall that probably cause the median pontine infarct; **b** a lateral wall plaque that probably cause the lateral pontine infarct. (T2WI)

A plaque was identified if there was eccentric wall thickening, whereas the thinnest part was estimated to be <50% of the thickest point by visual inspection [6]. All cross-sections with eccentric plaque were classified based on their plaque orientation being centered on the ventral, dorsal, and lateral (left or right) sides of the vessel (Fig. 1). Each cross-section was grouped into 1 of the 4 quadrants. In cases when the plaque was distributed more than 1 quadrant, the quadrant with the maximal plaque thickness was chosen [2]. For each patient, we calculated the total length of BA atherosclerosis plaques (plaque length = plaque slices × slice thickness + slices gaps). A culprit plaque was defined if it fulfilled the following criteria: 1, an infarct occurred in the pon (Fig. 2); 2, the BA plaque can be seen on the same slice of the infarct or on the adjacent upper or lower slice; 3, there was a probable casual relationship between the plaque and the infarct (dorsal wall plaque with median pontine infarcts or lateral wall plaque for lateral pontine infarcts). All images were reviewed by two experienced readers blinded to clinical data, who used the software of the syngofast View-Viewer for DICOM images (Ver.1.0.0.34). The differences between the two observers were solved by consensus.

### Statistical analysis

**Fig. 3:**
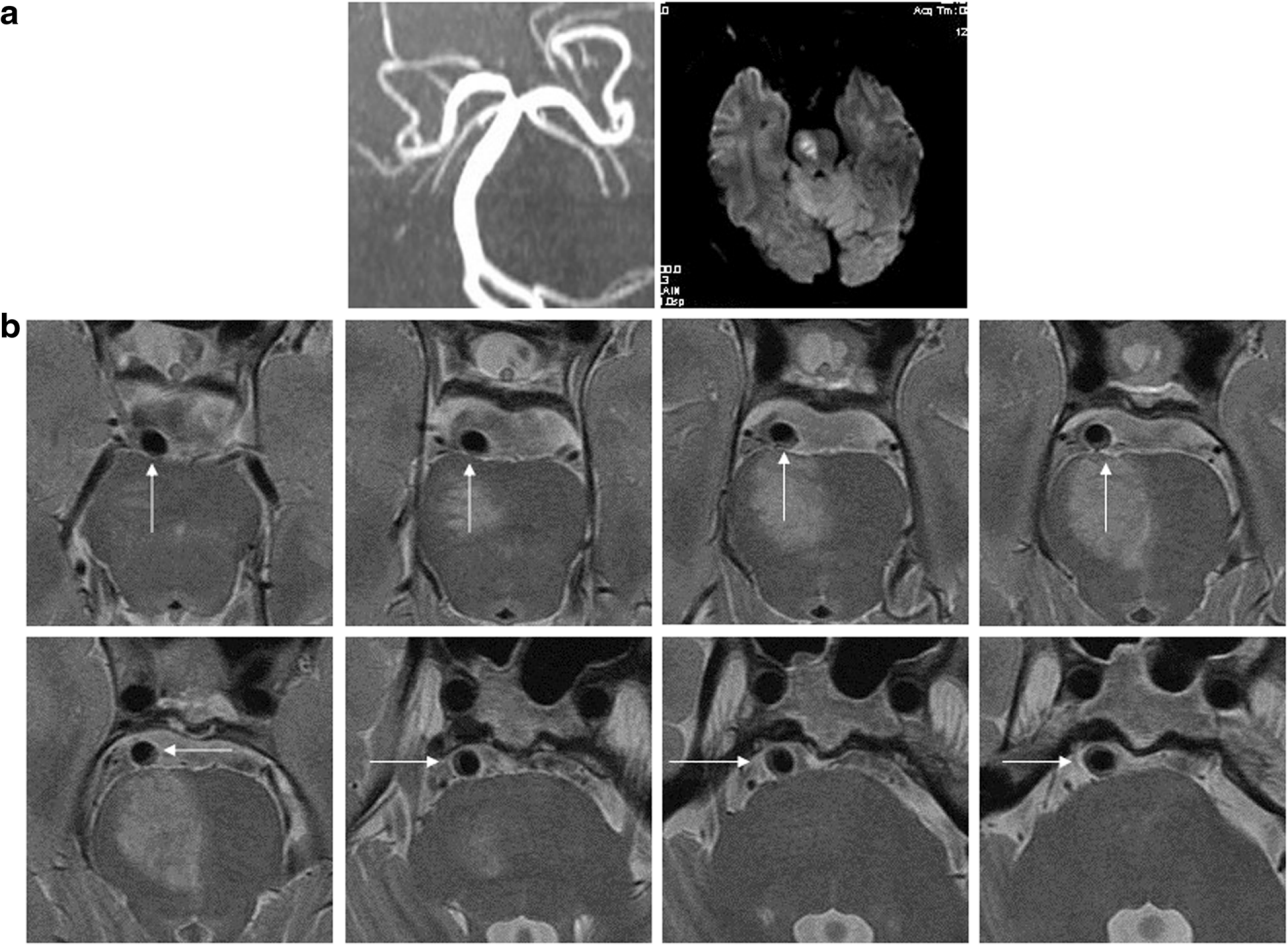
In a symptomatic BA stenosis (**a**), a plaque involving dorsal (4 slices) and lateral wall (4 slices) is seen on 8 of 12 consecutive T2WI slices (**b**, arrows on HR-MR; calculated percentage of plaque distribution: dorsal 50%, ventral 0%, and lateral 50%)

Quantitative data are expressed as mean ± SD and qualitative data are expressed as percentage. The continuous variables between the two groups were compared by the independent samples *t*-test. Categorical variables were compared by the chi-square test or Fisher exact test. For each stenosis, the percentage of individual plaque distribution was calculated (Fig. 3, Additional file 1: Table S1). The mean ventral, dorsal, lateral (left and right) plaque orientation of the total group was derived from the individual percentage distribution [3]. The comparison of the plaque distribution among different walls was performed by a Kruskal-Wallis test of the mean percentage of the distribution for each individual stenosis followed by Variance analysis and Bonferroni correction for multiple comparisons. Data comparisons between symptomatic and asymptomatic vessels were conducted with the Wilcoxon test. A probability value of <0.05 was considered statistically significant.

## Results

Seventy and four patients with BA plaque were considered for enrollment. Five patients with poor image qualities and eight patients with vertebral artery stenosis (≥50%) were excluded. Sixty-one patients were finally included for analysis. Twenty-five patients were symptomatic and thirty-six patients asymptomatic. Of the symptomatic patients, 18 had pontine infarcts, 2 had infarcts involving both pontine and extra-pontine area (1 with thalamus infarct and 1 with cerebellum infarct), and 5 with extra-pontine infarcts (1 with cerebellum infarct, 1 with occipital lobe infarct, and 3 with thalamic infarct). Of the asymptomatic patients, 14 had silent pontine infarcts. Table 1 summarizes the demographic data, showing no difference between symptomatic and asymptomatic patient groups.

**Table 1:** Demographic and clinical data

	Symptomatic BA *n* = 25	Asymptomatic BA *n* = 36	P
Age, years	66 ± 9	60 ± 12	0.192
Male	19 (76.0%)	23 (63.9%)	0.404
Hypertension	14 (56.0%)	19 (52.8%)	1.000
Hyperlipidemia	15 (60.0%)	20 (55.6%)	0.796
Diabetes	13 (52.0%)	17 (47.2%)	0.797
Smokers	17 (68.0%)	23 (63.9%)	0.790
Stenosis degree	34 ± 10%	35 ± 9%	0.809

A total of 752 HR-MRI image slices were reviewed. Plaques were identified in 305 slices. Plaque distribution was found to be eccentric in 299 (98.0%) and concentric in 6 (1.97%) cross sections. Only seven patients (11.5%) had a one-slice plaque, while most BA plaque involved multiple slices on HR-MRI. The average length of BA atherosclerosis plaques was 12.16 ± 5.61mm (10.30 ± 6.44mm in symptomatic and 13.46 ± 7.03mm in asymptomatic patients, *p* = 0.079).

The plaques distributed at 1 quadrant of BA wall on 271 (88.9%) image slices, 2 quadrants on 18 (5.9%) slices, 3 quadrants on 10 (3.3%) slices, and 4 quadrants on 6 (1.97%) slices. Thirty -nine patients only with 1 quadrant of BA plaque were found, 16 patients with 2 quadrants, 7 patients with 3 quadrants, 4 patients with 4 quadrants. The percentage of plaque distribution was similar in ventral (29.0%), dorsal (37.6%) and lateral walls (33.1%; *P* > 0.05, Kruskal-Wallis test). However, in symptomatic group, plaques were more frequently located at the dorsal (42.5%) and lateral (41.2%) walls than at the ventral walls (16.1%; *P* < 0.05, Variance analysis and Bonferroni correction test; Table 2). Compared with symptomatic patients, asymptomatic patients more likely had their plaques distributed at the ventral walls (*P* = 0.022). Symptomatic patients with pontine infarcts had more “culprit plaques” (17/20, 85.0%) than the patients with silent pontine infarcts (2/14, 14.3%; *p* = 0.000, Table 3).

**Table 2:** Basilar Artery Plaque Distribution

Vessels	Ventral Wall	Dorsal Wall	Lateral Wall	*P* *
All patients (61)	29.0%	37.6%	33.1%	1.000
Symptomatic (25)	16.1%	42.5%	41.2%	<0.05^a^
Asymptomatic (36)	38.0%	34.3%	27.4%	1.000
P	0.022	0.318	0.06	

P indicates comparisons between symptomatic and asymptomatic, P*indicates comparisons in the ventral, dorsal, and lateral sides of BA wall

^a^ventral vs. Dorsal, *p* = 0.009; ventral vs. Lateral, *p* = 0.013; dorsal vs. lateral *p* = 1.000

**Table 3:** Culprit plaque in symptomatic and silent pontine infarcts

Patients	Culprit plaque	Non-culprit plaque	Total
Symptomatic	17 (85.0%)	3 (15.0%)	20
Silent pontine infarcts	2 (14.3%)	12 (85.7%)	14

χ2 = 16.703, *p* = 0.000

## Discussion

In this study, it was observed low-grade BA plaques had a long distribution and evenly involved ventral, dorsal and lateral walls. They did not follow the rule of coronary artery and middle cerebral artery atheroscleros is that plaques tend to locate at the opposite side of the orifices of penetrating arteries. However, BA plaques in symptomatic patients predominantly distributed in the dorsal and lateral walls. Comparatively, BA plaques in asymptomatic patientsmore likely located at ventral walls. Interestingly, it was also observed that the culprit plaques were only associate with symptomatic infarcts, not with silent infarcts. These results suggest the plaque distribution of BA atherosclerosis has unique characteristics and is closely relevant with ischemic events.

There have been several HR-MRI studies focusing on symptomatic BA atherosclerosis [7–11]. Klein et al. enrolled 41 patients with pontine infarct. More than 70% patients had a BA plaque detected on HR-MRI, including a high proportion of patients with normal basilar angiograms. The authors hypothesized BA plaques might protrude into the orifice of the perforators and cause the infarcts [7]. In another study, 38 symptomatic patients with at least 30% stenosis were recruited [8]. The plaque distribution on the narrowest lumen slice was evaluated. The authors reported the ventral wall was more likely involved. The current study has several strengths. First, the whole plaque distribution of BA was evaluated and the culprit plaques were further defined based on vascular anatomy. Second, both symptomatic and asymptomatic patients were included, which make it possible to perform a comparative analysis. Third, only the patients with low grade BA stenosis and without vertebral artery stenosis were enrolled, that make the mechanism of embolism less likely account for the pontine infarcts.

The underlying pathophysiology of BA distribution is intriguing. Pathologic studies have shown that early atherosclerotic plaques appear to develop at the sites with the low or oscillatory wall shear stress [12]. Such sites commonly locate at the outer wall of a bifurcation, the inner wall of a curved artery, and the apex of a confluence. The blood flow of BA comes from two vertebral arteries and enters posterior cerebral arteries, with many penetrating arteries arise from its dorsal and lateral wall. Given the more widely and complicated microanatomy of BA than coronary artery and middle cerebral artery, it is difficult to identify the low sheer stress zones by simply following the rules established in coronary artery atherosclerosis. Further computerized hemodynamic studies on BA are warranted.

The results of our study are clinically meaningful. First, about two-thirds of BA plaques locate at ventral and dorsal walls, nearby the penetrating artery orifices, suggesting BA atherosclerosis has a high risk of developing penetrating infarcts. It may partly explain why intracranial stenting therapy has more snow-plowing complications in patients with BA atherosclerosis than the patients with middle cerebral artery atherosclersosis [3, 13, 14]. The close association between culprit plaques and symptomatic pontine infarcts suggest the knowledge of BA plaque distribution may potentially help us to estimate the likelihood of occlusion of the penetrating arteries and reduce the complication risk during stenting. Second, the underlying mechanisms of brain stem silent infarcts have been less well studied. Potential etiologies were presumed to be BA atherosclerosis or small vessel disease [11]. The high prevalence of non-culprit plaques in patients with silent pontine infarcts in our study doesn’t support the parent artery plaque as the causes. These plaques are more likely innocent bystander although they can be a potential marker for unhealthy vessel tree conditions.

Our study suffered from several limitations. First, the pathophysiology difference between low-grade and advanced BA stenosis has been not well studied. In current study, a proportion (7/25) of extra-pontine ischemic lesions were observed in symptomatic patients, suggesting the mechanisms of embolism may also involve. The interpretations of our data should be cautious and may not be applicable in advanced BA atherosclerosis. Second, we can not entirely exclude the possibility that the acute cerebral infarcts were caused by the branches or penetrating arteries lesions in our patients. Third, the retrospective design and the small sample size might lead to selective bias and thus limited its reliability.

## Conclusion

In this study, we described the plaque distribution of low-grade BA plaque and reported its clinical relevance. Further prospective studies are required to investigate whether our findings are helpful in stratifying stroke risk in patients with BA atherosclerotic disease.

## Additional file

Additional file 1: Table S1. Percentage of individual plaque distribution. Calculations of the percentage of individual plaque distribution: The quadrant grouping slice is patient specific. For each stenosis, the percentage of individual plaque distribution was calculated. If a stenosis only had one slice with ventral plaque, for example, the percentage of its individual plaque distribution is ventral 100%, dorsal 0%, latera l0%. If the stenosis have 3 slices (ventral 2, dorsal 1), the percentage is ventral 66%, dorsal 33%, lateral 0% (DOCX 19 kb)

## Abbreviations

3D TOF: 3Dimensional Time-of-flight
BA: Basilar artery
FOV: Field of view
HR-MRI: High-resolution magnetic resonance imaging
PDWI: Proton density weighted imaging
T1WI: T1 weighted imaging
T2WI: T2 weighted imaging
