# Supplementary material for: Plaque distribution of low-grade basilar artery atherosclerosis and its clinical relevance
Source: BMC Neurol. 2017 Jan 10;17:8. doi: 10.1186/s12883-016-0785-y (PMC5223551; doi:10.1186/s12883-016-0785-y)
Supplement: Additional file 1: Table S1. — Percentage of individual plaque distribution. Calculations of the percentage of individual plaque distribution: The quadrant grouping slice is patient specific. For each stenosis, the percentage of individual plaque distribution was calculated. If a stenosis only had one slice with ventral plaque, for example, the percentage of its individual plaque distribution is ventral 100%, dorsal 0%, latera l0%. If the stenosis have 3 slices (ventral 2, dorsal 1), the percentage is ventral 66%, dorsal 33%, lateral 0% (DOCX 19 kb) [file 12883_2016_785_MOESM1_ESM.docx]

**Table S1 Percentage of individual plaque distribution**

|  | Plaque Slices | Ventral(%) | Dorsal(%) | Lateral(%) |
| --- | --- | --- | --- | --- |
| Symptomatic |  |  |  |  |
| 1 | 7 | 4(57%) | 0 | 3(42%) |
| 2 | 7 | 1(14%) | 0 | 6(85%) |
| 3 | 5 | 0 | 2(40%) | 3(60%) |
| 4 | 2 | 0 | 2(100%) | 0% |
| 5 | 4 | 0 | 2(50%) | 2(50%) |
| 6 | 1 | 0 | 0 | 1(100%) |
| 7 | 4 | 2(50%) | 0 | 2(50%) |
| 8 | 5 | 2(40%) | 1(20%) | 2(40%) |
| 9 | 8 | 3(37.5%) | 3(37.5%) | 2(25%) |
| 10 | 4 | 0 | 2(50%) | 2(50%) |
| 11 | 8 | 4(50%) | 4(50%) | 0 |
| 12 | 3 | 0 | 2(66%) | 1(33%) |
| 13 | 3 | 2(66%) | 1(33%) | 0 |
| 14 | 9 | 1(11%) | 4(44%) | 4(44%) |
| 15 | 3 | 0 | 2(67%) | 1(33%) |
| 16 | 4 | 0 | 2(50%) | 2(50%) |
| 17 | 5 | 1(20%) | 1(20%) | 3(60%) |
| 18 | 2 | 0 | 2(100%) | 0 |
| 19 | 1 | 0 | 1(100%) | 0 |
| 20 | 8 | 0 | 4(50%) | 4(50%) |
| 21 | 8 | 3(37.5%) | 2(25%) | 3(37.5%) |
| 22 | 5 | 1(20%) | 3(60%) | 1(20%) |
| 23 | 1 | 0 | 0 | 1(100%) |
| 24 | 1 | 0 | 0 | 1(100%) |
| 25 | 1 | 0 | 1(100%) | 0 |
| Asymptoamtic |  |  |  |  |
| 26 | 6 | 1(16%) | 4(66%) | 1(16%) |
| 27 | 5 | 0 | 0 | 5(100%) |
| 28 | 1 | 0 | 1(100%) | 0 |
| 29 | 3 | 1(33%) | 1(33%) | 1(33%) |
| 30 | 10 | 4(40%) | 2(20%) | 4(40%) |
| 31 | 6 | 3(50%) | 2(33%) | 1(16%) |
| 32 | 3 | 0 | 2(66%) | 1(33%) |
| 33 | 2 | 0 | 0 | 2(100%) |
| 34 | 5 | 1(20%) | 4(80%) | 0 |
| 35 | 10 | 0 | 4(40%) | 6(60%) |
| 36 | 6 | 0 | 4(66%) | 2(33%) |
| 37 | 6 | 3(50%) | 0 | 3(50%) |
| 38 | 4 | 3(75%) | 1(25%) | 0 |
| 39 | 3 | 3(100%) | 0 | 0 |
| 40 | 4 | 1(25%) | 0 | 3(75%) |
| 41 | 5 | 0 | 4(80%) | 1(20%) |
| 42 | 5 | 3(60%) | 2(40%) | 0 |
| 43 | 10 | 10(100%) | 0 | 0 |
| 44 | 1 | 0 | 1(100%) | 0 |
| 45 | 4 | 3(75%) | 1(25%) | 0 |
| 46 | 3 | 0 | 3(100%) | 0 |
| 47 | 3 | 3(100%) | 0 | 0 |
| 48 | 3 | 0 | 2(66%) | 1(33%) |
| 49 | 10 | 8(80%) | 1(10%) | 1(10%) |
| 50 | 5 | 4(80%) | 0 | 1(20%) |
| 51 | 6 | 0 | 6(100%) | 0 |
| 52 | 8 | 0 | 1(12.5%) | 7(87.5%) |
| 53 | 4 | 2(50%) | 1(25%) | 1(25%) |
| 54 | 6 | 3(50%) | 3(50%) | 0 |
| 55 | 5 | 5(100%) | 0 | 0 |
| 56 | 7 | 3(42%) | 0 | 4(57%) |
| 57 | 8 | 7(87.5%) | 0 | 1(12.5%) |
| 58 | 7 | 6(86%) | 1(14%) | 0 |
| 59 | 11 | 3(27%) | 3(27%) | 5(45%) |
| 60 | 9 | 2(22%) | 5(55%) | 2(22%) |
| 61 | 2 | 0 | 0 | 2(100%) |
